# Ornamental motifs and visual concerns: Exploring aesthetic pleasure and conservation intention in architectural heritage renovation

**DOI:** 10.1371/journal.pone.0359522

**Published:** 2026-10-07

**Authors:** Han Zhang, Muhizam Mustafa, Zihao Cao, Yongchun Mao, Karima Hussein Abdulhaleem Hemida, Xiaomei Song

**Affiliations:** 1 School of the Arts, Universiti Sains Malaysia, Gelugor, Penang, Malaysia; 2 School of Arts and Design, Qilu University of Technology, Jinan, China; 3 School of Arts, Jeddah International College, Jeddah, Saudi Arabia; 4 College of Art Design, Yangzhou Polytechnic Institute, Yangzhou, China; Yunnan University, CHINA

## Abstract

In the ongoing efforts of architectural heritage renovation, ornamental motifs serve not only as aesthetic embellishments but also as carriers of cultural symbolism. However, how their visual perception influences public aesthetic judgments and conservation intentions remains underexplored. This study focuses on two types of culturally embedded motifs – the native Chinese dragon and the imported lion – and examines their impact under two visual forms (2D and 3D) within a 2 × 2 experimental framework. A combination of eye-tracking measures and questionnaire assessments was employed with a sample of 80 participants to investigate the effects of iconographic category and visual form on four dependent variables: aesthetic pleasure, conservation intention, fixation time, and time to first fixation. Results revealed significant main effects of both iconographic category and visual form across all outcome variables. Notably, significant interactions were found in conservation intention and time to first fixation. This study advances understanding of how iconographic meaning and visual form jointly shape visual attention, aesthetic evaluation, and conservation intention in architectural heritage contexts.

## Introduction

Within the current global agenda for the sustainable preservation of cultural heritage, architectural heritage is increasingly recognized not only as a repository of historical memory but also as a vital resource for promoting cultural sustainability. In alignment with UNESCO’s Recommendation on the 2011 Historic Urban Landscape and the 2030 Agenda for Sustainable Development, the practices of conservation and renovation have gradually shifted from a technocratic paradigm focused on material preservation toward an integrative approach that includes social participation, aesthetic reconfiguration, and cultural revitalization [1]. Renovation is no longer solely concerned with the physical continuity of structures; it has become a means of reconstructing cultural identity. Consequently, public cultural perception, emotional attitudes, and behavioral engagement are no longer peripheral concerns, but central components in shaping the sustainability of heritage conservation [2,3]. In this context, the public’s cognitive responses, affective orientations, and willingness to engage are emerging as critical driving forces behind sustainable architectural heritage conservation [4,5].

Architectural heritage renovation involves not only the preservation of overall structural integrity but also the expression of ornamental details, among which ornamental motifs constitute a core visual element that embodies rich cultural symbolism and aesthetic value. Such motifs often reflect the political order, religious beliefs, and identity construction of specific historical periods [6,7]. Notably, architectural ornamentation encompasses both indigenous cultural symbols, such as the dragon, and foreign elements, such as the lion, which was introduced via Buddhist transmission. These motifs collectively form a pluralistic iconographic system that reflects the historical processes of cultural fusion [8,9]. In addition, the visual form of these motifs, such as three-dimensional carvings versus two-dimensional pictorial renderings, produces significantly different effects in terms of visual perception and emotional arousal [10]. Yet it remains under-explored whether such formal differences further influence public aesthetic evaluations and attitudes toward architectural heritage.

Although a growing body of research acknowledges the cultural value and communicative potential of ornamental elements in architectural heritage, most existing studies focus on historical interpretation, stylistic analysis, or conservation techniques [4–6,11]. Far less attention has been paid to how the public visually perceives such motifs and how these perceptual experiences shape their conservation intentions. Moreover, the potential interaction between different iconographic categories and visual forms in influencing public aesthetic response and attitudinal orientation lacks sufficient empirical investigation to date.

Building upon the above theoretical considerations, this study aims to investigate how iconographic category (native dragon versus imported lion) and visual form (2D versus 3D) influence public responses to architectural heritage ornamentation. Specifically, the study examines their effects on visual attention (fixation time and time to first fixation), aesthetic pleasure, and conservation intention using a 2 × 2 eye-tracking experiment combined with questionnaire measures. This study focuses on ornamental motifs in Chinese architectural heritage and examines public responses under controlled experimental conditions. It does not attempt to evaluate architectural heritage as a whole or real-world restoration projects. By integrating objective visual behavior with subjective evaluations, this study seeks to provide empirical evidence for heritage renovation strategies that balance cultural symbolism and visual communication.

## Literature review

### Ornamental motifs in Chinese architecture

Ornamental motifs in traditional Chinese architecture carry profound historical and aesthetic significance, deeply rooted in cultural symbolism and philosophical thought. These motifs are not merely ornaments; rather, they embody evolving philosophical concepts, social meanings, and historical narratives that have developed over centuries [6,12]. For instance, architectural ornamentation in China has been shown to transcend physical form by conveying literary and sociopolitical meanings, reflecting the ideological structures of premodern Chinese society [13]. In particular, the historical evolution of specific motifs highlights the integration between physical continuity and symbolic representation [14]. The aesthetic value of ornamental motifs in traditional Chinese architecture stands in stark contrast to the modernist architectural movement’s rejection of ornamentation [15]. As many contemporary architects have observed, while modernist critique often regards ornament as superfluous, traditional Chinese architectural aesthetics demonstrate how motifs function as indispensable carriers of cultural meaning and value [11].

The cultural origins of ornamental motifs give rise to distinctive symbolic meanings. For example, the dragon, originating from indigenous Chinese culture, has long functioned as a national totem symbolizing supreme authority and mastery over natural forces such as wind and rain. It is traditionally associated with imperial power and auspiciousness [16]. In contrast, the lion motif is a foreign symbol that entered China through the transmission of Buddhist art, later becoming localized as a guardian figure symbolizing conservation, elite status, and blessings of peace and prosperity [8]. These motifs have been deeply embedded in architectural design as carriers of cultural narratives and as elements that enhance aesthetic appeal. In traditional Chinese architecture, dragon and lion motifs coexist and often appear side by side. The dragon continues to signify ritual authority and imperial benevolence, while the lion, through a process of cultural adaptation, has come to represent domestic security and spiritual safeguarding. Lions are frequently depicted together with dragons and phoenixes, contributing to a layered visual language of blessing, hierarchy, and conservation [9]. This iconographic coexistence reflects the interweaving of Confucian, Buddhist, and folk beliefs, enriching the symbolic system that underpins ornamental traditions in Chinese architectural heritage [6,17].

There are notable perceptual differences between two-dimensional (2D) and three-dimensional (3D) visual forms of architectural ornamentation. 3D motifs, due to their depth cues and variations in light and shadow, tend to increase visual complexity, an attribute that is often associated with heightened aesthetic pleasure and arousal levels [10]. The choice between 2D and 3D forms usually depends on the intended impact and contextual demands of the architectural project [18]. Eye-tracking studies have shown that both 2D and 3D motifs can effectively capture visual attention when they are color-rich or morphologically complex. In contrast, minimally decorated facades tend to lack visual salience and focal interest [19]. While 3D ornamentation offers a more immersive and tactile experience, 2D motifs remain vital in architectural design, as they convey essential elements of cultural aesthetics and can engage viewers through compositional structure and symbolic meaning [20]. Moreover, the integration of ornamental elements in architecture plays a key role in shaping spatial perception, redirecting visual attention, and influencing first impressions of a space [21].

Recent years have seen a growing body of eye-tracking research examining how architectural patterns, decorative elements, and façade details guide visual attention and aesthetic judgement [22]. Existing studies have employed a wide range of ornamental stimuli, including repetitive geometric patterns [15], symbolic figurative motifs [23], and façade-level decorative compositions [24], to investigate differences in gaze behavior and perceptual processing. However, much of this literature prioritizes formal visual properties such as complexity, density, and contrast, while devoting limited attention to the cultural origins and symbolic meanings embedded in ornamental motifs. This gap indicates the need for experimental designs that not only control visual form but also explicitly distinguish ornamental motifs according to their cultural symbolism, thereby enabling a more nuanced understanding of how visual attention and conservation-related attitudes are jointly shaped [25].

### Architectural heritage conservation

The conservation of architectural heritage emphasizes achieving a balance between preserving historical integrity and meeting contemporary demands for sustainability and usability [1]. Restoration efforts often involve the coexistence of old and new architectural elements, requiring careful consideration of both cultural meanings and physical characteristics without compromising practical needs [26]. In addition, the conservation of modern architectural heritage, often characterized by rationalism, functionalism, and industrialized materials, faces distinct challenges. The absence of clear consensus on criteria for restoration and preservation has led to the need for integrative approaches that reconcile conflicting demands [27]. In recent years, heritage conservation has gradually shifted from an expert-led model to one that incorporates broader public engagement. Public perception and attitudes are increasingly recognized as key psychological mechanisms driving the sustainability of heritage conservation [4,28]. Research has demonstrated that individuals’ cultural identification, aesthetic experiences, and levels of historical knowledge significantly influence their intentions to support heritage conservation [3,15].

Driven by intangible cultural factors, the public often forms emotional connections to heritage through processes of symbolic interpretation [29]. As cultural symbols, ornamental motifs possess the potential to evoke identity-based resonance and stimulate conservation motivation, making them valuable tools for heritage communication and education. However, despite their rich cultural connotations, ornamental motifs in architectural heritage are frequently marginalized in actual conservation practices. In many restoration and renovation projects, such motifs are treated as non-essential embellishments, optional elements that are often simplified, substituted, or removed altogether [30,31]. Moreover, the widespread commodification and symbolic appropriation of traditional motifs in consumer products have led to a weakening, and in some cases distortion, of their cultural significance [32]. This phenomenon underscores the importance of enhancing public understanding of the cultural attributes embedded in ornamental motifs. Such awareness is a critical precondition for fostering conservation intention and achieving more holistic approaches to heritage preservation.

In recent years, conservation intention has garnered increasing attention as a key psychological variable for assessing public engagement and support in cultural heritage preservation. Existing studies have shown that visitors’ perceptions of authenticity, the depth of their cultural experiences, and their sense of place attachment significantly enhance their willingness to support heritage conservation [33]. Furthermore, overall visitor satisfaction, particularly the emotional quality of the viewing experience, has been confirmed as closely related to subsequent conservation tendencies [34]. Attitudinal factors are also widely recognized as strong predictors of protective behavior; individuals who hold positive evaluations of heritage and regard it as personally meaningful are more likely to engage in conservation activities [5]. These findings are consistent with the Theory of Planned Behavior (TPB), which posits that behavioral intentions are jointly influenced by attitudes, subjective norms, and perceived behavioral control [5,35]. In this light, measuring public conservation intentions toward ornamental motifs in architectural heritage can help predict future behavioral responses and provide a theoretical basis for the design of cultural communication strategies and policy interventions.

### Eye-tracking experiment

Eye-tracking technology plays a critical role in investigating how individuals perceive visual information [36,37]. Historically, eye-tracking was first applied in disciplines such as geography, cartography, and landscape studies to examine how people observe and process complex spatial data [38,39]. With technological advancement, researchers have come to recognize that eye-tracking data reveal participants’ attentional preferences and the distribution of visual attention, making the method increasingly popular in fields such as design, visual aesthetics, and environmental perception [40,41]. Moreover, eye-tracking also assists heritage experts in understanding how people visually distinguish between the original and renovated elements of architectural heritage, thus informing more effective management and conservation strategies. By uncovering patterns of visual engagement, this method can help evoke deeper resonance among contemporary audiences in heritage contexts [42].

Today, eye-tracking technology has emerged as an effective tool in the study of architectural heritage, offering insights into how individuals view and interact with historical buildings and monuments. This technique enables researchers to track the eye movements of observers, generating data on visual behavior that can inform conservation and restoration strategies [43]. For instance, Rusnak and Szewczyk [44] employed eye-tracking to evaluate the renovation of historic sites, using fixation time and fixation count as indicators to assess differences in visual attention before and after architectural interventions. Similarly, response time has been used as a judgment metric and found that architects demonstrated shorter time to first fixation when viewing images of building facades, suggesting that physical attributes of architectural design can be manipulated to influence aesthetic experiences [45]. Despite these advances, research remains limited on how people perceive ornamental motifs in architectural heritage through visual processing, particularly in relation to differences in motif form and their impact on conservation intention. Eye-tracking has yet to be fully utilized to investigate the interplay between visual form variation and attitudinal outcomes in the context of ornamental elements.

Fixation time and time to first fixation are commonly used indicators for understanding visual attention. Fixation time refers to the amount of time an observer spends fixating on a specific location, typically measured in seconds (s). Research has shown that fixation duration varies depending on the cognitive demands of a visual scene, with more challenging visual conditions slowing down processing speed [46,47]. Longer fixation times generally indicate deeper cognitive processing; when viewers spend more time fixating on a particular area, it suggests they are engaging with complex information or that the region is perceived as more interesting or relevant [48]. Time to first fixation refers to the duration it takes for a viewer to fixate on a specific area of interest (AOI) from the moment visual observation begins, also measured in seconds. A shorter time to first fixation indicates that the stimulus is more salient or visually prominent [49,50].

In architectural heritage studies, ornamental motifs have been classified through multiple typological perspectives [7,15]. From a contemporary image-based clustering perspective, ornaments can be categorized according to material, colour, and geometric configuration [51]. In terms of formal structure, ornamental motifs have also been grouped into five categories: animal, vegetal, anthropomorphic, totemic, and geometric patterns [52]. However, from an experimental standpoint, not all ornamental categories are equally suitable for controlled visual analysis. Abstract geometric and vegetal motifs often lack stable and widely shared symbolic meanings, which may increase variability in individual interpretation [53]. By contrast, figurative ornaments, particularly zoomorphic motifs, exhibit strong cultural codification and high visual recognizability [54,55], making them more appropriate for examining perceptual and attitudinal responses in eye-tracking research. Within this category, the dragon and the lion represent two coexisting yet culturally differentiated elements in Chinese architectural heritage. The dragon originates from indigenous cosmological traditions, whereas the lion was introduced through cross-cultural transmission and subsequently localized [9]. This contrast allows cultural origin to be operationalized as an independent variable, while two-dimensional and three-dimensional representations are employed to manipulate visual form [19]. Illustrated stimuli were adopted to ensure strict control over non-essential visual variables, thereby isolating the effects of iconographic category and visual form [25].

### Theoretical foundation

The conservation of architectural heritage has garnered increasing attention for its role in shaping identity and ensuring cultural continuity. In everyday life, individuals often engage with architectural heritage over extended periods, and their cognitive responses to such visual stimuli serve as important indicators for evaluating the potential for sustainable heritage preservation [15,56]. Eye-tracking, as an effective tool for studying visual cognition, has been validated in both architectural heritage and design-related research, particularly when combined with subjective measures for more comprehensive analysis [22,57]. Therefore, when investigating aesthetic evaluation and conservation intention in the context of architectural heritage, focusing on visual cognition offers a theoretically coherent and methodologically promising research direction.

While research on architectural heritage has traditionally focused on sustainable conservation and historical value, the role of architectural aesthetics in public reception and heritage dissemination should not be overlooked. According to aesthetic response theory [58], aesthetics is not merely an outcome-based judgment, but a critical component of cultural symbol processing. Recent studies on image-based social media platforms have shown that users’ attitudes toward heritage architecture are significantly shaped by visual appeal, suggesting that aesthetic attraction is a key driver of public engagement and heritage communication [59]. At the urban scale, architectural aesthetics also exhibit tangible economic effects, visual ambiance in historic districts has been linked to housing price premiums exceeding 20%, underscoring the monetary value of heritage aesthetics [60]. Consequently, aesthetic impact assessment has become an increasingly important aspect of heritage renovation projects, ensuring that the results not only preserve cultural identity but also resonate with public appreciation [61]. In particular, if the ornamental design of a building’s façade is mishandled, it may undermine both its visual recognition and economic appeal [62]. This further emphasizes the need to prioritize aesthetic evaluation in the conservation and adaptive reuse of ornamental motifs in architectural heritage.

The Theory of Planned Behavior (TPB) offers a useful framework for understanding how attitudes, subjective norms, and perceived behavioral control influence individuals’ intentions and actions [35]. This theory is particularly relevant when examining how aesthetic interventions in historic architecture affect conservation intention. According to TPB, intention, considered the immediate antecedent of behavior, is shaped by three components: attitude, subjective norm, and perceived behavioral control [5]. In the context of architectural heritage conservation, aesthetic enhancements can strengthen conservation intention by influencing the public’s attitude toward the heritage site. A positive aesthetic experience can increase public recognition and emotional attachment to heritage architecture, thereby motivating protective intentions [63]. In our study, TPB is employed to assess participants’ attitudes toward architectural renovation and, more specifically, to measure their intention to engage in heritage conservation. This includes evaluating the extent to which individuals feel capable and willing to take part in actions related to the conservation of specific architectural heritage sites. The goal is to investigate the relationship between different approaches to architectural renovation and the resulting levels of conservation intention.

This study simultaneously examines the interaction between visual form and iconographic category in relation to aesthetic evaluation, visual attention, and conservation intention in architectural heritage. Compared to existing research, which often focuses on a single aesthetic factor or conservation strategy [15,59,64], our study adopts a more integrative approach to explore the complex relationships among these variables. This approach not only captures subtle differences in visual attention among audiences but also quantifies their emotional and cognitive responses to heritage architecture, thereby offering deeper insights into variations in aesthetic pleasure and conservation motivation. Our findings contribute to a more nuanced understanding of how to balance historical authenticity with contemporary aesthetics and provide empirical evidence to support heritage conservation strategies that simultaneously enhance public engagement.

## Methods

This study employed a controlled 2 × 2 between-subjects factorial experimental design, combining eye-tracking measures with quantitative questionnaire assessments. An eye-tracking device was used to capture and record participants’ physiological eye movements as they viewed images of architectural heritage. The experimental design followed a 2 (Iconographic Category: native culture dragon vs. imported culture lion) × 2 (Visual Form: 2D vs. 3D) factorial structure. The dependent variables included fixation time, time to first fixation, aesthetic pleasure, and conservation intention.

### Experimental stimuli

To minimize the influence of extraneous visual variables commonly present in real-world photographs, the study employed custom-designed images as experimental stimuli. This approach enabled precise operationalization of the independent variables while maintaining strict experimental control.

A standardized 3D model of a traditional Chinese architectural arch gate was constructed using SketchUp. This gate form was selected because it represents a widely recognizable heritage architectural typology and provides a symmetrical and stable structural framework for the placement of ornamentation (Fig 1). To ensure cross-condition consistency, all structural parameters, including proportions, dimensions, orientation, roof form, and decorative framework, were held constant across all stimuli. The base model was then rendered using Enscape to generate high-resolution images with uniform lighting conditions, neutral material textures, and simplified backgrounds, thereby reducing unintended visual salience outside the target ornamental area.

**Fig 1:**
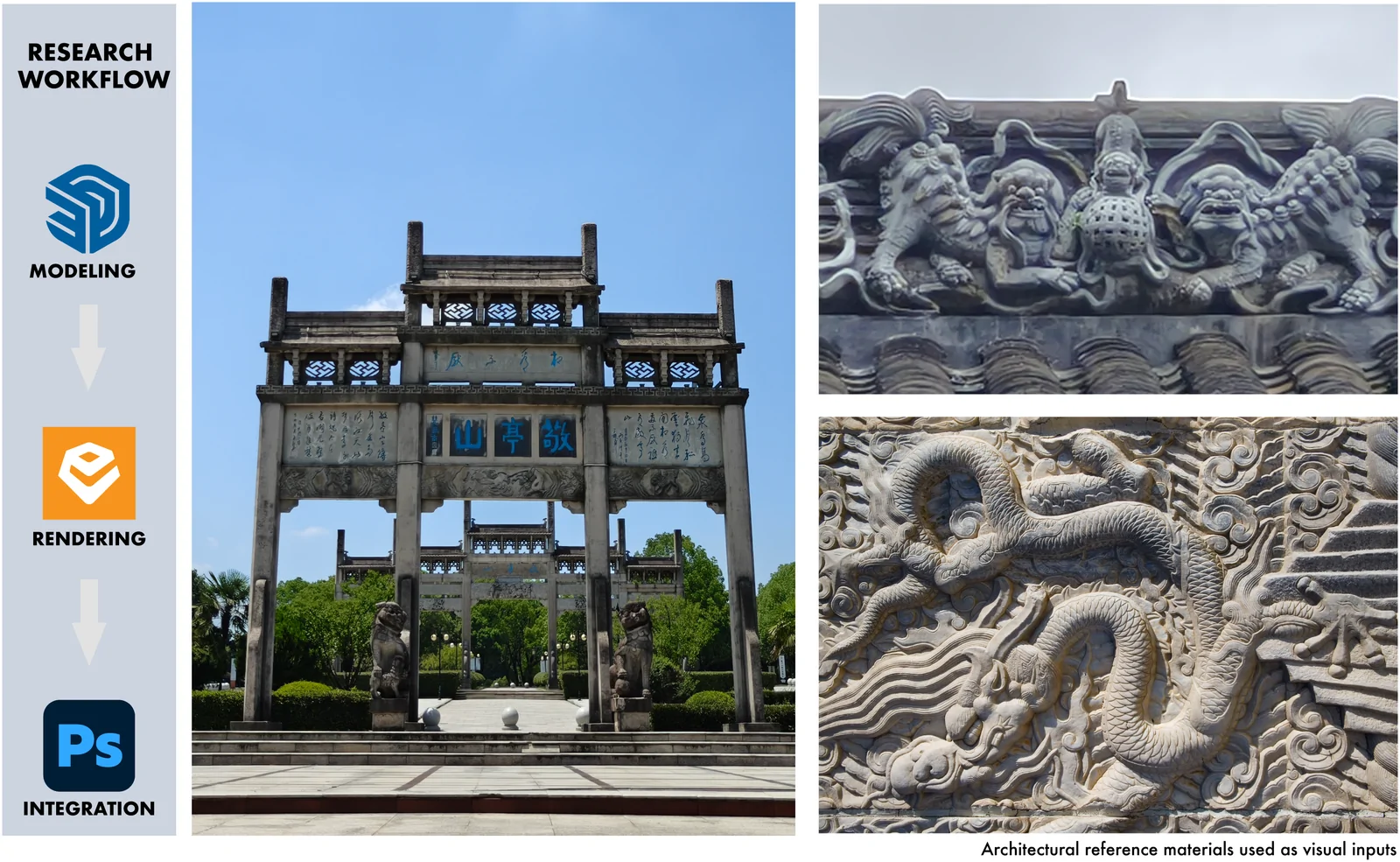
The arch gates and the carved depictions of dragons and lions found in real-world architectural heritage. The images by the third author.

On this controlled architectural base, ornamental motifs were systematically embedded using Adobe Photoshop to operationalize the two independent variables, namely iconographic category and visual form. With respect to iconographic category, dragon and lion motifs were selected to represent indigenous and imported cultural origins, respectively, while maintaining comparable scale, placement, and functional roles within the architectural composition. In terms of visual form, each motif was designed in both 2D and 3D versions. The 2D motifs were presented as flat, relief-like graphics without depth cues, whereas the 3D motifs incorporated volumetric modelling, shadow casting, and consistent perspective to enhance perceived depth and materiality.

To isolate the visual processing of ornamental motifs, Areas of Interest (AOIs) were defined consistently across all stimuli, corresponding to the central decorative panels where the motifs were positioned (Fig 2). Outside the AOIs, all architectural elements were visually identical, ensuring that any observed differences in eye-tracking metrics and subjective evaluations could be attributed solely to variations in iconographic category and visual form. The final stimulus set comprised four images: A1 (lion, 3D), A2 (dragon, 3D), A3 (lion, 2D), and A4 (dragon, 2D). These four conditions constituted a 2 × 2 factorial design.

**Fig 2:**
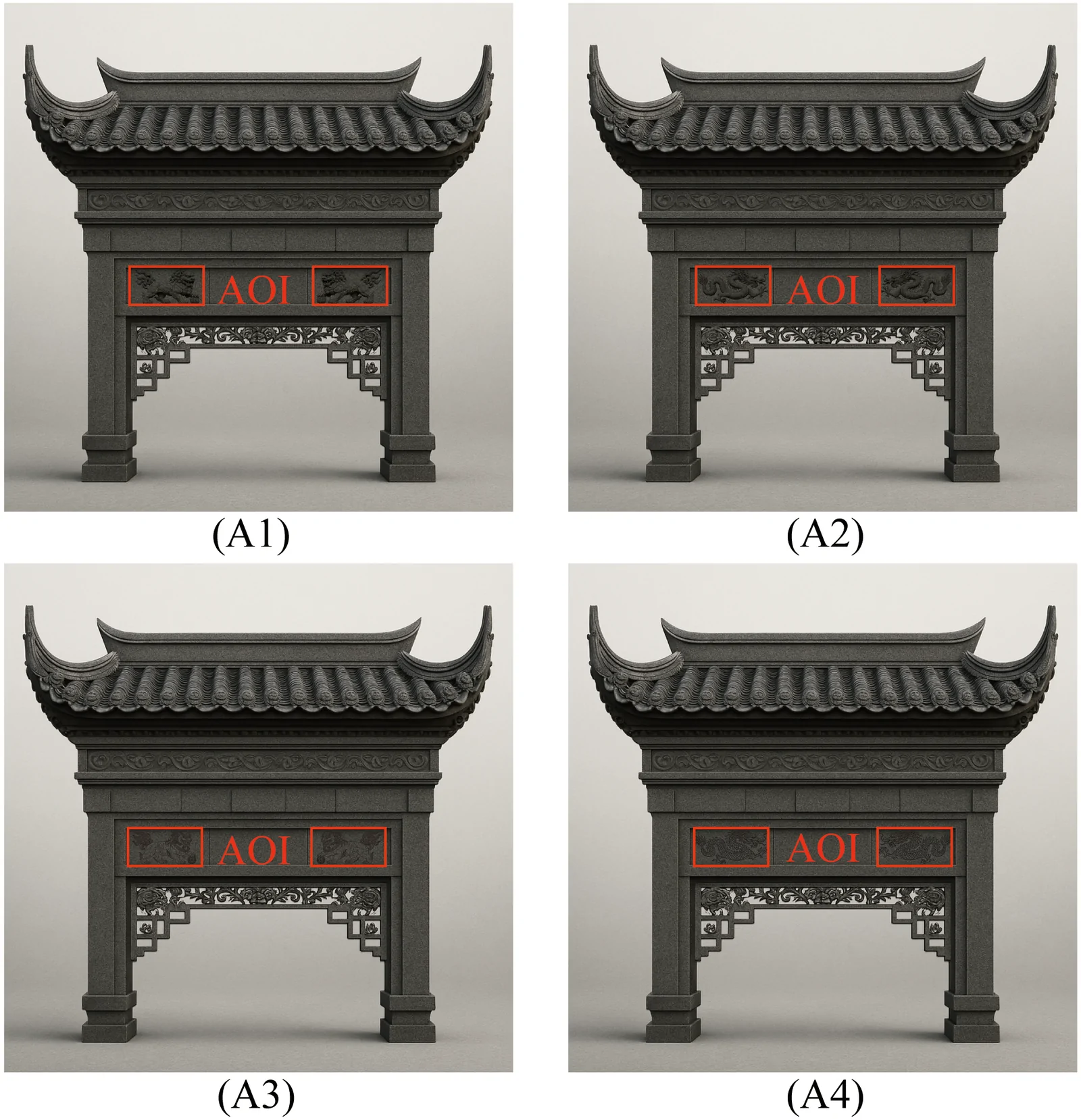
Stimulus image design for the experiment. The images by the third author.

### Participant

A total of 98 participants were recruited in Yangzhou, China, all of whom had no professional background in heritage-related fields. Participants were recruited from local universities and public platforms. This sampling strategy was adopted to minimize the potential for homogeneity in expert knowledge or domain-specific biases. The participants were adults with undergraduate or master's level educational backgrounds, working across a variety of sectors, including private business management, finance, food service, and healthcare. The deliberate inclusion of non-experts was intended to reflect general public perspectives, thereby ensuring that the findings would be more broadly applicable to public engagement contexts rather than restricted to professional debates. Such sampling helps control for potential bias caused by prior domain knowledge or entrenched expert attitudes [65].

During data collection, participants with eye-tracking data sampling rates below 90% were excluded. All participants reported no prior experience with eye-tracking experiments. The final valid sample consisted of 80 participants, including 38 males and 42 females, aged between 18 and 28 years. These participants had normal or corrected-to-normal vision ranging from 1.0 to 1.5 and reported no symptoms of color blindness or visual impairments, ensuring that visual differences would not confound the results. Informed consent was obtained from all participants before the experiment, and each received CNY 40 as compensation upon completion.

### Instrument

This study employed the Tobii Pro Glasses 2 eye-tracking system as the primary data collection tool, as it provides detailed and objective measures of visual attention, revealing participants’ authentic reactions to different architectural elements. Compared to traditional methods such as surveys or interviews, eye-tracking technology allows for the direct quantification of visual behavior and the capture of participants’ initial attentional allocation and visual preferences during aesthetic evaluation. This approach is particularly well-suited for examining participants’ immediate responses to materials and ornamentation in architectural heritage, minimizing subjective bias and offering more reliable data to explore the relationship between visual attention and conservation intention. During the experiment(Fig 3), participants wore the Tobii Pro Glasses 2 while viewing the experimental stimuli presented on the monitor. The system continuously recorded participants’ gaze positions throughout the viewing process, from which fixation time (FT) and time to first fixation (TTFF) were extracted to quantify visual attention toward the predefined areas of interest (AOIs).

**Fig 3:**
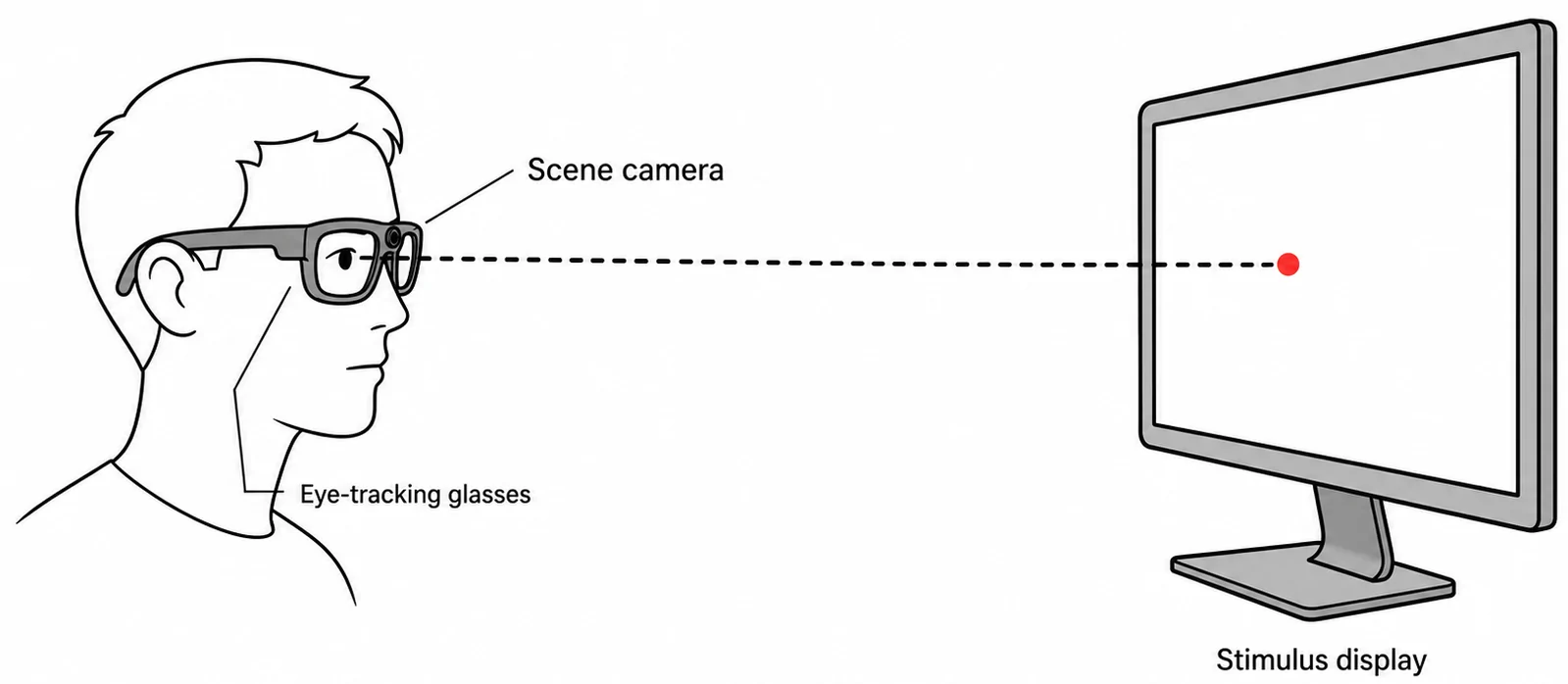
Eye-tracking apparatus and experimental setup using Tobii Pro Glasses 2. The images by the first author.

The experiment was conducted in a closed, soundproof laboratory to ensure environmental consistency and precision in data collection. Stimulus images were imported into the device interface, and participants were instructed to wear the eye-tracking glasses during the experiment. The stimuli were presented on a 27-inch LCD flat-screen monitor with a resolution of 3840 × 2160 and a refresh rate of 160 Hz. Eye-tracking data were recorded using the Tobii Pro Glasses Controller software (Tobii Pro, Stockholm, Sweden), and subsequently processed and exported using Tobii Pro Lab software (Tobii Pro, Stockholm, Sweden).

We employed a structured, closed-ended questionnaire to collect quantitative data on participants’ conservation intention and aesthetic pleasure toward the four architectural heritage stimuli (Table 1). The conservation intention scale was adapted from Li et al. [33] and consisted of three items. Participants were asked to rate each item on a 5-point Likert scale, ranging from 1 (“strongly disagree”) to 5 (“strongly agree”). The aesthetic pleasure scale was adapted from a previously validated instrument with an available Chinese version [66]. The subject matter was modified to fit the context of this study, and the revised scale was pilot-tested before formal data collection. The complete bilingual questionnaire is provided in the Supporting Information (S1 File).

**Table 1:** The items in the questionnaire used.

Variable	Item
Aesthetic pleasure	I like to look at this arch gate.
	This is an attractive arch gate.
	This arch gate is nice to see
	This is a beautiful arch gate.
Conservation Intention	I think this arch gate inspires my desire for cultural conservation.
	I think that after seeing this arch gate, I will promote the idea of heritage conservation to others.
	After seeing this arch gate, I think that adopting heritage preservation behaviors is meaningful.

To ensure the reliability of the measures, we conducted a pilot study before the formal experiment. Based on the preliminary data, we adjusted the experimental procedure and refined the questionnaire to enhance internal consistency and construct validity. Since the study was conducted in China and all participants were native Chinese speakers, the questionnaire was translated into Chinese and pre-tested. A total of 65 respondents from three residential communities participated in the pilot survey. The Cronbach’s alpha for the conservation intention scale was 0.82, and for the aesthetic pleasure scale it was 0.88, indicating high internal consistency. Exploratory factor analysis (EFA) was conducted on all seven items to examine the structural validity of the questionnaire. Principal Axis Factoring with Varimax rotation was used. The Kaiser-Meyer-Olkin (KMO) measure was 0.81, and Bartlett’s test of sphericity was significant (χ²(21) = 236.78, p < .001), confirming the suitability of the data for factor analysis and supporting the construct validity of the scales.

The four dependent variables were measured using two complementary instruments. Eye-tracking technology was used to capture the objective visual attention indicators, namely fixation time (FT) and time to first fixation (TTFF), whereas a structured questionnaire was employed to assess the subjective outcome variables of aesthetic pleasure and conservation intention.

### Data collection

Data were collected from June 25 to July 30, 2024, at the Eye-Tracking Laboratory of Yangzhou Polytechnic Institute. Before the experiment began, all participants were given a detailed briefing on the study procedure to ensure full comprehension of the experimental steps, thereby enhancing the reliability and validity of the data. After providing written informed consent, participants were assisted by the researcher in properly wearing the eye-tracking equipment, followed by a calibration procedure. During this process, no information regarding the stimuli was disclosed to the participants; instead, they were simply instructed to freely observe all visual content while keeping their heads relatively stable. To measure participants’ visual attention to different Areas of Interest (AOIs) within the stimulus images, two key eye-tracking indicators were recorded: Fixation Time (FT) and Time to First Fixation (TTFF). These two metrics provided quantifiable measures of how participants allocated visual attention across different AOIs throughout the viewing session, particularly in response to different types of visual information.

The experimental procedure is illustrated in Fig 4. Participants were randomly assigned to one of four groups, each corresponding to one of the four stimuli. Seated in front of the screen, participants were first asked to read the on-screen instructions at their own pace. Once they confirmed their understanding, they proceeded by pressing the spacebar. A central fixation point was then displayed for 1000 milliseconds, followed by the automatic presentation of the experimental stimulus [67]. During the stimulus observation phase, participants were instructed to carefully view the image while simultaneously completing two rating scales. These scales measured their responses on aesthetic pleasure and conservation intention. Upon completing all ratings, participants pressed the spacebar again to end the experimental session.

**Fig 4:**
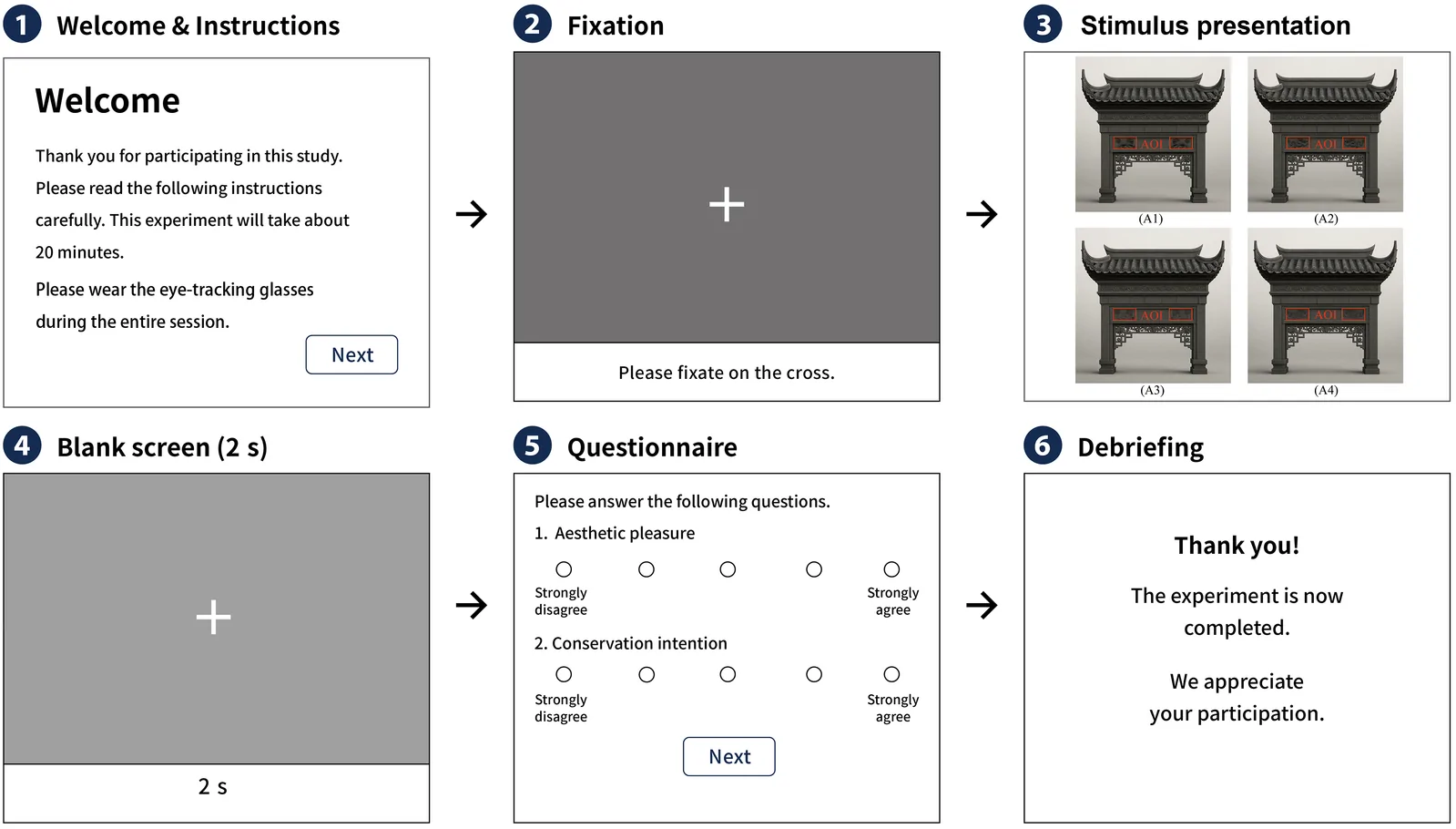
Experimental procedures. The images by the first author.

### Data analysis

Raw data for fixation time and time to first fixation were directly extracted from Tobii Pro Lab (Tobii Pro, Stockholm, Sweden). Numerical data for aesthetic pleasure and conservation intention were manually entered into Excel to ensure accurate recording of subjective ratings and prepare the dataset for subsequent statistical analysis. All statistical analyses were conducted using SPSS for Windows, version 26.0 [68]. Initially, descriptive statistics were used to summarize the results for the four experimental conditions across all dependent variables, including fixation time, time to first fixation, aesthetic pleasure, and conservation intention [69].

To examine the effects of the independent variables, Iconographic Category and Visual Form, on the dependent variables (fixation time [s], time to first fixation [s], aesthetic pleasure, and conservation intention), a series of two-way ANOVAs were conducted. Both main effects and interaction effects were analyzed to explore the influence of each factor. Main effects analysis assessed the independent impact of each variable (e.g., Iconographic Category) on the outcome measures, allowing us to determine how each factor individually influenced fixation time, time to first fixation, aesthetic pleasure, and conservation intention. When a significant interaction was detected, we conducted further simple effects analysis to explore the interaction patterns in greater detail. Simple effects analysis enabled us to examine the effect of one independent variable at specific levels of the other [70]. For example, in the case of a significant interaction between Iconographic Category and Visual Form on conservation intention, comparisons were made between native culture dragon and imported culture lion under the 2D condition, as well as between the two under the 3D condition. All experimental procedures involving human participants were reviewed and approved by the Academic Committee of Yangzhou Polytechnic Institute (Approval No. YPI-2024-HED-032) and were conducted in accordance with the Declaration of Helsinki. All participants were adults who voluntarily participated and provided written informed consent before the experiment, and all data were collected and analyzed anonymously to ensure privacy and confidentiality. The minimal dataset underlying all analyses is provided in S1 Dataset.

## Results

### Conservation intention

The results for conservation intention are presented in Table 2. A significant main effect of Visual Form was found, F(1, 76) = 16.443, p < 0.001, ηp² = 0.178. There was also a significant main effect of Iconographic Category, F(1, 76) = 41.938, p < .001, ηp² = 0.356. Moreover, a significant interaction effect between Iconographic Category and Visual Form was observed (Fig 5), F(1, 76) = 8.092, p = .006, ηp² = 0.096.

**Table 2:** Results of Conservation Intention.

Visual Form	Iconographic Category				Total	
	Native culture dragon		Imported culture lion			
	Mean (M)	Standard Deviation (SD)	Mean (M)	Standard Deviation (SD)	Mean (M)	Standard Deviation (SD)
2D	3.03	0.58	2.53	0.70	2.78	0.69
3D	3.98	0.43	2.70	0.71	3.34	0.87
Total	3.50	0.70	2.62	0.70	3.06	0.83

**Fig 5:**
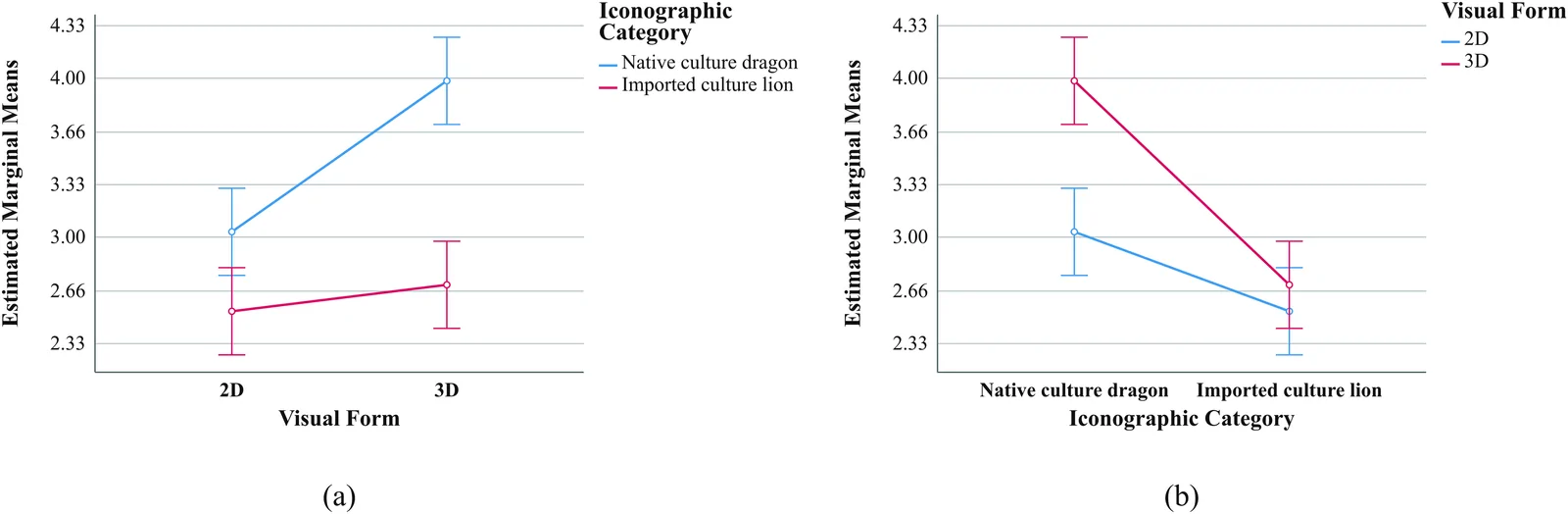
Interaction effect on conservation intention: (a) Visual Form on the x-axis, with lines representing Iconographic Category; (b) Iconographic Category on the x-axis, with lines representing Visual Form.

Simple effects analysis results are shown in Table 3. For the native culture dragon, conservation intention was significantly lower in the 2D condition than in the 3D condition. For the imported culture lion, there was no significant difference in conservation intention between the 2D and 3D conditions. In the 2D condition, the native culture dragon elicited significantly higher conservation intention than the imported culture lion. Similarly, in the 3D condition, conservation intention for the native culture dragon remained significantly higher than that for the imported culture lion.

**Table 3:** Simple effects in Conservation intention.

Interaction	Variables	I	J	Mean difference (I-J)	F	p	ηp²
Iconographic Category × Visual Form	dragon	2D	3D	-2.850	23.802	<0.001	0.238
	lion	2D	3D	-0.500	0.733	0.395	0.010
	2D	dragon	lion	1.500	6.593	0.012	0.080
	3D	dragon	lion	3.850	43.436	<0.001	0.364

### Aesthetic pleasure

The results for aesthetic pleasure are shown in Table 4. A significant main effect of Visual Form was found, F(1, 76) = 36.171, p < 0.001, ηp² = 0.322. A significant main effect of Iconographic Category was also observed, F(1, 76) = 4.940, p = .029, ηp² = 0.061. However, the interaction between Iconographic Category and Visual Form was not statistically significant, F(1, 76) = 1.709, p = .195, ηp² = 0.022.

**Table 4:** Results of Aesthetic pleasure.

Visual Form	Iconographic Category				Total	
	Native culture dragon		Imported culture lion			
	M	SD	M	SD	M	SD
2D	3.24	0.50	2.90	0.43	3.07	0.49
3D	3.69	0.43	3.60	0.33	3.64	0.38
Total	3.46	0.51	3.25	0.52	3.36	0.52

### Fixation time

The results for fixation time are presented in Table 5. A significant main effect of Visual Form was found, F(1, 76) = 46.442, p < 0.001, ηp² = 0.379. A significant main effect of Iconographic Category was also observed, F(1, 76) = 4.887, p = .030, ηp² = 0.060. The interaction effect between Iconographic Category and Visual Form was not statistically significant, F(1, 76) = 3.709, p = .058, ηp² = 0.047.

**Table 5:** Results of Fixation time.

Visual Form	Iconographic Category				Total	
	Native culture dragon		Imported culture lion			
	M	SD	M	SD	M	SD
2D	11.93	1.72	11.79	2.87	11.86	2.34
3D	16.32	2.35	14.24	1.87	15.28	2.35
Total	14.13	3.01	13.01	2.69	13.57	2.89

### Time to first fixation

The results for time to first fixation are presented in Table 6. A significant main effect of Visual Form was found, F(1, 76) = 117.075, p < 0.001, ηp² = 0.606. A significant main effect of Iconographic Category was also observed, F(1, 76) = 12.566, p < 0.001, ηp² = 0.142. The interaction effect between Iconographic Category and Visual Form was statistically significant (see Fig 6), F(1, 76) = 6.952, p = .010, ηp² = 0.084.

**Table 6:** Results of time to first fixation.

Visual Form	Iconographic Category				Total	
	Native culture dragon		Imported culture lion			
	M	SD	M	SD	M	SD
2D	2.43	0.48	3.05	0.46	2.74	0.56
3D	1.62	0.37	1.71	0.46	1.66	0.41
Total	2.03	0.59	2.38	0.82	2.20	0.73

**Fig 6:**
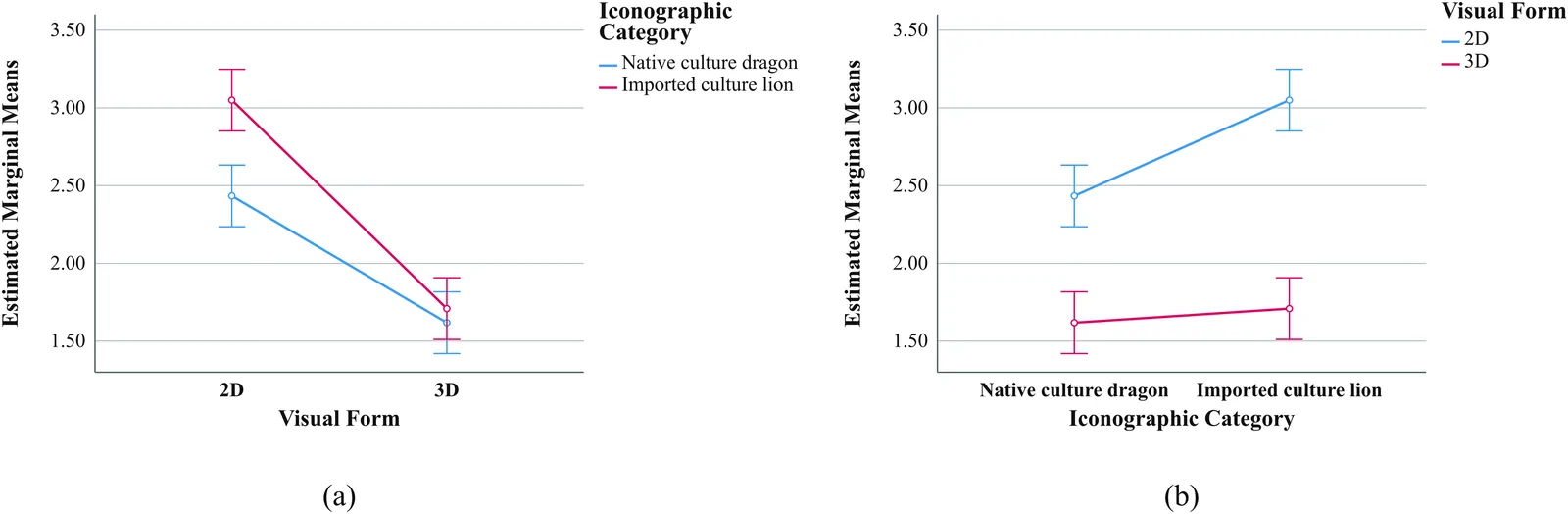
Interaction effect on time to first fixation: (a) Visual Form on the x-axis, with lines representing Iconographic Category; (b) Iconographic Category on the x-axis, with lines representing Visual Form.

Simple effects analysis for time to first fixation is shown in Table 7. For the native culture dragon, time to first fixation was significantly longer in the 2D condition compared to the 3D condition. Similarly, for the imported culture lion, participants took longer to fixate in the 2D condition than in the 3D condition. In the 2D condition, the native culture dragon took a shorter time to first fixation than the imported culture lion. However, in the 3D condition, no significant difference in time to first fixation was observed between the native culture dragon and the imported culture lion.

**Table 7:** Simple effects in Time to first fixation.

Interaction	Variables	I	J	Mean difference (I-J)	F	p	ηp²
Iconographic Category × Visual Form	dragon	2D	3D	0.816	33.484	<0.001	0.306
	lion	2D	3D	1.341	90.542	<0.001	0.544
	2D	dragon	lion	−0.616	19.105	<0.001	0.201
	3D	dragon	lion	−0.090	0.412	0.523	0.005

## Discussion

### Interpretation of differences in conservation intention

This study found that both Iconographic Category and Visual Form exerted significant main effects on participants’ conservation intention toward architectural heritage, and a significant interaction effect was also observed. Overall, participants reported significantly higher conservation intention for the native cultural motif, the dragon, compared to the imported lion, supporting the “ingroup preference” effect proposed by cultural identity theory. That is, individuals who grow up within a shared cultural narrative are more likely to exhibit internalized identification responses when encountering culturally congruent symbols such as the dragon [5]. Importantly, this difference extends beyond mere cognitive recognition of symbolic meaning; it reflects the transformative potential of symbolic identification into behavioral intention. In other words, a strong sense of cultural belonging can be externalized as a motivational drive to protect specific heritage elements [71]. At the same time, 3D motifs elicited significantly stronger conservation intention than 2D ones, suggesting that visual form, as a perceptual entry point, enhances both aesthetic processing depth and emotional arousal. This finding supports a “perceptually mediated protection” pathway in which sensory engagement facilitates attitudinal and behavioral alignment with heritage conservation.

The significant main effect of Visual Form further highlights the active role of sensory channels in shaping cultural attitudes. The finding that 3D motifs significantly enhanced conservation intention suggests that images with greater spatial depth and formal complexity may elicit richer perceptual processing and more nuanced symbolic interpretation [10]. This aligns with aesthetic response theory, which posits that visual–tactile synesthetic imagination can intensify psychological engagement with cultural objects [72,73]. Compared to 2D motifs, 3D representations are more likely to evoke a sense of immersion and ritualistic association, transforming the image from something merely visible into something worthy of conservation [19]. However, this result also prompts critical reflection: in the context of heritage restoration or digital reconstruction, has too much emphasis been placed on delivering visual impact, potentially at the expense of conveying the deeper symbolic meaning and educational value of the motifs themselves?

Most importantly, this study revealed a significant interaction effect between Iconographic Category and Visual Form, suggesting that cultural symbolism and formal expressiveness do not operate independently but rather function in tandem to activate public attitudinal responses [74]. Under the condition of the native cultural motif, the dragon, the 3D format significantly increased conservation intention. This implies that only when cultural identification and perceptual engagement are simultaneously activated can conservation intention reach its peak. In contrast, for the imported motif, the lion, even when presented in 3D form, the symbolic resonance remained insufficient to elicit a comparable attitudinal response. This “threshold effect of symbolism” illustrates that in visual communication for heritage, enhancing visual impact alone (e.g., through three-dimensional rendering) cannot compensate for a lack of cultural semantic resonance [75]. The visual expressiveness of an image must work in concert with its symbolic depth in order to meaningfully shape public perceptions of its protective worthiness [76].

### Interpretation of differences in aesthetic pleasure

Both Iconographic Category and Visual Form significantly influenced participants’ aesthetic pleasure. Participants assigned higher aesthetic ratings to the native dragon motif than to the imported lion motif. This difference suggests that aesthetic responses to ornamental motifs may be associated not only with their visual characteristics but also with their culturally embedded meanings. The dragon occupies a prominent position in Chinese architectural and visual traditions, with its symbolic associations reinforced through repeated cultural representation [66,77]. This cultural familiarity may facilitate aesthetic appreciation by enhancing the motif’s recognizability and perceived cultural significance. At minimum, the present findings indicate that greater cultural familiarity was not associated with lower aesthetic pleasure in this experiment [78]. Instead, the native dragon motif showed a modest aesthetic advantage over the imported lion motif. Given the relatively small effect of Iconographic Category, however, this difference should be interpreted cautiously and cannot establish cultural familiarity or identification as the mechanism underlying the observed aesthetic preference.

In terms of visual form, 3D motifs received significantly higher aesthetic ratings than 2D motifs, confirming the crucial role of formal complexity and spatial dimensionality in the processing of visual aesthetics. Due to their enhanced structural expressiveness and depth, 3D motifs are more likely to elicit rich sensory associations and emotional responses from viewers. In architectural contexts in particular, qualities such as sculptural relief, tactile simulation, and material realism can amplify aesthetic pleasure [72]. From the perspective of aesthetic processing theories, 3D designs align more closely with the “complexity-integration” model, which favors efficient perceptual processing and activates the viewer’s “form exploration system,” ultimately leading to heightened subjective aesthetic responses. However, it is also important to recognize that the aesthetic advantage of 3D forms may be moderated by cultural context. In some cases, public expectations regarding architectural heritage aesthetics do not necessarily align with values such as complexity, dimensionality, or sculptural richness. Instead, they may shift depending on environmental settings, viewing habits, and culturally shaped visual literacy [79]. Therefore, while the aesthetic appeal of 3D motifs appears robust, their effective application must be interpreted within specific cultural and spatial contexts.

It is worth noting that no significant interaction effect was found between Iconographic Category and Visual Form in the context of aesthetic pleasure. This suggests that the two variables operate along relatively independent pathways in shaping aesthetic experience. This finding stands in sharp contrast to the results for conservation intention, where cultural identification and formal processing exhibited a clear synergistic effect. The divergence implies that aesthetic pleasure may not function as a prerequisite or antecedent for conservation motivation, but rather as a parallel and relatively autonomous psychological process. This challenges assumptions made in previous studies [59], which often implied that strong aesthetic appeal naturally translates into higher conservation motivation, and vice versa. Our findings caution against reducing “visual appeal” to the sole entry point for heritage communication. If the distinction between “beauty” and “value” in public perception is overlooked, there is a risk of overestimating the cultural efficacy of form-based design [15]. Therefore, future architectural heritage strategies should more systematically differentiate the functional boundaries between aesthetic enjoyment and cultural attitudes, avoiding the conflation of aesthetics with identification or visual form with symbolic meaning.

### Interpretation of differences in visual attention

In terms of visual attention, this study used fixation time (FT) and time to first fixation (TTFF) as core indicators. The results revealed significant main effects of both Iconographic Category and Visual Form on fixation time. Overall, participants spent significantly more time fixating on the native cultural motif, the dragon, than on the imported motif, the lion. This suggests that the symbolic significance of cultural motifs can effectively prolong visual engagement. This finding aligns with prior research on cultural identity theory, which posits that individuals allocate greater attentional and cognitive resources to symbols that resonate with their cultural identity and familiarity [80]. At the same time, 3D motifs also significantly increased fixation time. Compared to 2D motifs, 3D forms promoted more sustained viewing, likely due to their perceived complexity, spatial depth, and the viewer’s exploratory desire to process fine-grained visual details [21]. These results suggest that Visual Form not only influences aesthetic evaluation but also plays a direct and foundational role in shaping attentional allocation, serving as the initial perceptual gateway to public cultural experience.

Although the interaction effect for fixation time was not significant, the simple-effects analysis of TTFF revealed a distinct pattern during the early stage of visual attention. For both the dragon and lion motifs, participants exhibited significantly longer TTFFs in the 2D condition than in the 3D condition. This consistent effect suggests that 3D representations captured initial visual attention more rapidly than their 2D counterparts. The spatial depth, structural projection, and shadow gradients associated with 3D representations may provide stronger perceptual cues that facilitate gaze initiation [19]. In the 2D condition, however, the native dragon motif was fixated significantly earlier than the imported lion motif. This finding suggests that iconographic category may influence attentional priority when motifs are presented in a less spatially salient form. The greater cultural familiarity and recognizability of the dragon motif may have facilitated its earlier detection; however, these potential mechanisms were not directly assessed in the present study. Notably, this difference between the dragon and lion motifs was no longer evident in the 3D condition. This finding suggests that the enhanced perceptual salience of 3D representations may attenuate differences in initial attentional priority associated with iconographic category. These findings suggest that early visual attention reflects the joint influence of formal salience and motif-related characteristics, with their relative contributions varying across representational conditions.

It is particularly noteworthy that under the 3D condition, the TTFF difference between the dragon and lion motifs disappeared. This suggests that when visual form becomes sufficiently salient, differences between ornamental motifs at the level of attentional onset are partially neutralized. In high-intensity visual conditions, the visual system appears to prioritize structural features over symbolic attributes [81]. This finding offers a dual implication for the visual communication of cultural heritage. On one hand, 3D form can serve as a universal strategy for capturing initial visual attention, functioning as an effective perceptual hook. On the other hand, if the goal is to convey cultural meaning or activate identity-related responses, relying solely on 3D form is insufficient [20]. In other words, the mechanism of visual onset and the mechanism of cognitive elaboration are not inherently aligned, and designers must be deliberate about the perceptual goal of each stage. In heritage communication practices, visual strategies should move from “attention capture” to “immersive engagement,” and from “form-driven appeal” to “meaning-oriented decoding.” Without this progression, even if visual stimuli succeed in holding attention, they may ultimately fail to connect audiences to the deeper layers of cultural significance [82].

### Limitations and future recommendations

Despite the study’s effective use of experimental design to reveal the influence of Iconographic Category and Visual Form on aesthetic pleasure, visual attention, and conservation intention, several limitations should be acknowledged. First, the participant sample was relatively homogeneous, consisting of individuals with similar educational backgrounds and cultural experiences, which may limit the generalizability of the findings to broader populations. Since cultural background plays a critical role in interpreting iconographic symbols and attributing heritage value, the absence of cross-cultural comparisons or controls for cultural familiarity may have constrained the interpretation of cognitive responses, particularly toward the “lion” motif. Second, while the visual stimuli were derived from authentic architectural elements, they were static images presented against simplified backgrounds, which limited the simulation of the immersive spatial atmosphere typically encountered in real heritage environments. This simplification may have attenuated the tactile imagination and embodied perception normally associated with 3D forms. Third, although eye-tracking served as a valuable objective measure, the study focused only on fixation duration and time to first fixation, without incorporating more nuanced indicators such as scan paths or revisit frequency. Moreover, no physiological data (e.g., skin conductance, EEG) were collected to capture the emotional dimension of aesthetic or cultural engagement. Finally, conservation intention was assessed via self-reported questionnaires, which may be subject to social desirability bias, and no behavioral follow-up was conducted to establish a causal relationship between intention and actual conservation-related actions.

Building on the above reflections, future research can further expand and deepen the current findings along several key directions. First, it is recommended to incorporate cross-cultural samples or manipulate cultural familiarity conditions to investigate perceptual differences in how iconographic categories are interpreted across cultural contexts. This would help clarify the culturally moderated pathways linking identification, preference, and conservation intention. Second, the design of stimulus materials can be enhanced through the integration of virtual reality (VR) or 360-degree immersive imagery, allowing for a more realistic simulation of spatial and lighting conditions within heritage architecture. Such techniques would improve the perceived authenticity and experiential engagement associated with 3D motifs. Third, future studies should incorporate more granular eye-tracking metrics (e.g., scan path analysis, revisitation patterns, pupil dilation), along with physiological measures of emotional arousal, to construct dynamic interaction models between visual behavior and subjective perception. Fourth, longitudinal or intervention-based designs are encouraged to explore whether aesthetic pleasure exerts delayed effects or functions through mediating mechanisms in shaping conservation intentions, thereby advancing causal validation of the “visual processing – cognitive understanding – behavioral motivation” chain. Finally, considering that ornamental motifs are socially constructed cultural symbols, future research could combine text analysis, semantic priming, or in-depth interviews to examine how visual stimuli and interpretive meaning co-construct the public’s understanding of heritage significance.

## Conclusion

By combining eye-tracking measures with subjective questionnaire responses, this study systematically examined how symbolic meaning and perceptual design influence public responses to architectural heritage ornamentation. The findings demonstrated that both iconographic category and visual form significantly affected conservation intention, aesthetic pleasure, and visual attention. Specifically, native dragon motifs generated significantly higher conservation intention than imported lion motifs (F(1, 76) = 41.938, p < 0.001, ηp² = 0.356), suggesting that culturally embedded symbols can strengthen heritage-related behavioral motivation. Visual form also showed a substantial influence, with 3D representations producing higher conservation intention than 2D representations (F(1, 76) = 16.443, p < 0.001, ηp² = 0.178) and enhancing visual engagement through longer fixation time (F(1, 76) = 46.442, p < 0.001, ηp² = 0.379). In addition, 3D motifs received higher aesthetic pleasure ratings than 2D motifs (M = 3.64 vs. 3.07), F(1, 76) = 36.171, p < 0.001, ηp² = 0.322. They also showed shorter TTFFs than 2D motifs (M = 1.66 s vs. 2.74 s), F(1, 76) = 117.075, p < 0.001, ηp² = 0.606. Moreover, a significant interaction between iconographic category and visual form was identified for conservation intention (F(1, 76) = 8.092, p = 0.006, ηp² = 0.096), indicating that cultural symbolism and perceptual expression jointly shape heritage-related attitudes. However, aesthetic pleasure and conservation motivation did not follow an identical pattern. Although 3D motifs generally received higher aesthetic evaluations, the relationship between visual attractiveness and conservation intention was not simply linear. These findings suggest that aesthetic appreciation and heritage protection motivation represent related but partially distinct psychological processes.

The primary contributions of this study unfold across both theoretical and practical dimensions. Theoretically, this research is the first to introduce a dual-dimensional interaction paradigm, Iconographic Category vs. Visual Form, into the field of visual communication in architectural heritage. This framework bridges a critical gap in the existing literature by empirically examining the relationship between the symbolic attributes and formal expressions of ornamental motifs. The findings expand the applicability of aesthetic response theory within the context of cultural heritage and offer a cognitive psychological foundation for understanding the mechanisms that link visual attention, aesthetic pleasure, and conservation intention. Practically, the study provides actionable insights for heritage exhibition design, architectural motif restoration, and cultural product development. By revealing the advantages of 3D motifs in directing visual attention and activating conservation attitudes, and by identifying symbolic strength differences across cultural motifs, the research suggests that future heritage displays should not only enhance perceptual salience but also deepen cultural interpretation, thereby avoiding the pitfalls of form-driven, unidirectional messaging. Furthermore, the study highlights the importance of recognizing and responding to the dynamic relationship between cultural familiarity and perceptual receptivity, fostering a synergistic resonance between visual engagement and cultural preservation. These insights contribute to the ongoing re-cognition and re-activation of architectural heritage in contemporary restoration practice.

## Supporting information

S1 File Questionnaire scale. The bilingual questionnaire used to measure aesthetic pleasure and conservation intention.
(DOCX)

S1 Dataset Minimal dataset. The participant-level data underlying the analyses, including visual form, iconographic category, fixation duration, time to first fixation, conservation intention, and aesthetic pleasure.
(XLSX)

S1 Checklist Inclusivity in global research questionnaire. (DOCX)
