# Supplementary material for: Ornamental motifs and visual concerns: Exploring aesthetic pleasure and conservation intention in architectural heritage renovation
Source: PLoS One. 2026 Oct 7;21(10):e0359522. doi: 10.1371/journal.pone.0359522 (PMC13645022; doi:10.1371/journal.pone.0359522)
Supplement: Supplementary file 1 [file pone.0359522.s001.docx]

**标准化量表 -Standardized scales**

**审美愉悦**

**Aesthetic pleasure**

1. 我喜欢注视这座门楼

1. I like to look at this arch gate.

A.非常不同意 B. 不同意 C. 一般 D. 同意 E. 非常同意

A. strongly disagree B. disagree C. generally D. agree E. strongly agree

2. 这是一座具有吸引力的门楼

2. this is an attractive arch gate.

A.非常不同意 B. 不同意 C. 一般 D. 同意 E. 非常同意

A. strongly disagree B. disagree C. generally D. agree E. strongly agree

3. 这座门楼看起来不错

3. this arch gate is nice to see

A.非常不同意 B. 不同意 C. 一般 D. 同意 E. 非常同意

A. strongly disagree B. disagree C. generally D. agree E. strongly agree

4. 这是一座漂亮的门楼

4. this is a beautiful arch gate.

A.非常不同意 B. 不同意 C. 一般 D. 同意 E. 非常同意

A. strongly disagree B. disagree C. generally D. agree E. strongly agree

**保护意图**

**Conservation Intention**

1. 我认为这座门楼激发了我对文化保护的渴望

1. I think this arch gate inspires my desire for cultural conservation.

A.非常不同意 B. 不同意 C. 一般 D. 同意 E. 非常同意

A. strongly disagree B. disagree C. generally D. agree E. strongly agree

2. 我认为看完这座门楼，我会向其他人宣传遗产保护的理念

2. I think that after seeing this arch gate, I will promote the idea of heritage conservation to others.

A.非常不同意 B. 不同意 C. 一般 D. 同意 E. 非常同意

A. strongly disagree B. disagree C. generally D. agree E. strongly agree

3. 看完这座门楼，我认为采取遗产保护行为是有意义的

3. After seeing this arch gate, I think that adopting heritage preservation behaviors is meaningful

A.非常不同意 B. 不同意 C. 一般 D. 同意 E. 非常同意

A. strongly disagree B. disagree C. generally D. agree E. strongly agree
